# Efficacy, Safety, and Overall Quality of Life of Endoscopic Submucosal Dissection for Early Colorectal Cancer in Elderly Patients

**DOI:** 10.1155/2017/2386291

**Published:** 2017-07-03

**Authors:** Liang Liu, Xingjie Shen, Jingyu Zhu

**Affiliations:** Department of Gastroenterology, Ji'nan Central Hospital Affiliated to Shandong University, Ji'nan 250012, China

## Abstract

**Purpose:**

Studies reporting the treatment of early colorectal cancer (ECC) by endoscopic submucosal dissection (ESD) in elderly patients are lacking in China. The aim was to evaluate the efficacy, safety and overall quality of life of elderly patients with ECC who undergoing ESD.

**Methods:**

Three hundred and seventy-nine patients with 401 colorectal lesions entered into our study from March 2013 to March 2016 (Patients with an age 70 years old or older were divided into the elderly group and those who were less than 70-year-old entered the non-elderly group).

**Results:**

No significant differences were found in sex ratio, body mass index, location, endoscopic classification, pathological pattern, lesion size, mean procedure time, hospitalization days, complete excision, and en bloc resection rate between the two groups (*P* > 0.05). No significant differences were observed between the groups in terms of complications during and after ESD procedure (*P* > 0.05). There were no statistical differences between two groups in Quality of life index (QL-Index) and European Organization for Research and Treatment quality of life version 3.0 questionnaire (EORTC QLQ-C30) scores (*P* > 0.05).

**Conclusion:**

ESD was relatively safe and effective for elderly patients with ECC, and it may be an recommended first-line treatment.

## 1. Introduction

Colorectal cancer (CRC) remains one of the common malignant tumours worldwide. In recent years, the incidence of CRC in China is increasing, and it is expected that the incidence and mortality will be rising in the near future, severely impairing people's health and quality of life (QOL) [1, 2].

Early colorectal cancer (ECC) is defined as colorectal cancer which confined to the mucosa or submucosa, irrespective of the presence of regional lymph node metastases. Surgical operation is the mainstay in the management of CRC. However, the incidence of chronic disease may increase with increasing age; elderly patients with systemic diseases, such as diabetes and high blood pressure, together with poor gastrointestinal function, might not be able to endure such severe surgical trauma [3]. Endoscopic submucosal dissection (ESD) has been proven to be an effective therapeutic method for ECC, achieving the same curative effects [3, 4]. Unfortunately, studies reporting the treatment of ECC by ESD in elderly patients are lacking in China.

Thus, the aim of this study was conducted to investigate the safety, efficacy, and overall quality of life about the ESD in elderly patients with ECC.

## 2. Materials and Methods

### 2.1. Participants

This retrospective study was carried out from March 2013 to March 2016 in the Digestive Endoscopy Center of Ji'nan Central Hospital Affiliated to Shandong University, Ji'nan, China. A total of 401 colorectal lesions in consecutive 379 patients, all of whom underwent ESD for ECC, were included in the study. The study protocol was approved by the ethics committee for clinical research in our hospital.

All patients were provided written and informed consent prior to ESD. Patients with severe or refractory hypertension, heart disease, diabetes, liver disease, respiratory disease, and active bleeding were excluded from the study. Prior to the ESD, all patients had undergone examinations, including endoscopic ultrasonography, chromoendoscopy, thoracoabdominal computed tomography, and biopsy. All ESD procedures were performed by two experienced endoscopists.

Patients with an age 70 years old or older were divided into the elderly group (113 lesions in 109 patients), and those who were less than 70 years old entered the nonelderly group (288 lesions in 270 patients).  Table 1 shows the clinicopathological characteristics of the patients.

### 2.2. ESD Procedure and Postoperative Management

Anticoagulant and antiplatelet drugs were discontinued at least 7 days before ESD procedure. We treated each lesion separately; if patients with multiple lesions, they were received scheduled treatments on different days. Bowel preparation was based on intaking of four to five liters of polyethylene glycol prior to the ESD procedure. All patients underwent conscious sedation using of intravenous administration midazolam and pethidine hydrochloride prior to the operation.

First of all, narrow band imaging (NBI) endoscopy was used to observe the range of the lesion, and we marked the lesion with argon plasma coagulation (APC). Then, we raised the submucosa through submucosal injection. After that, a circumferential incision was performed by using a HOOK knife. Circumferential incisions were performed in order to lift the lesion, and all lesion dissections were operated by using IT knife. After removing the lesions, specimens were retrieved with forceps or basket. The margins were carefully investigated to ensure complete lesion resection before completing the operation [5]. Endoscopic preventive hemostasis for ulcer bed was performed by the APC or hot biopsy forceps. Hemostatic forceps or metal clips were used to manage intraoperative bleeding.

Patients without adverse events were permitted to drinking water the first day and eating soft food on the second day after the operation. Generally, patients enjoyed their normal diets on postoperative days 4-5. Patient's general condition and auxiliary examination results, such as defecation, abdominal signs, temperature, blood biochemical and routine examination, chest X-ray, abdominal plain film, and D-dimer, were recorded in order to determine if the occurrence of postoperative complications.

Follow-up colonoscopy and observation were performed at 3 and 6 months postoperatively. Moreover, we will have a long-time follow-up to all the patients.

### 2.3. Questionnaires

#### 2.3.1. Quality of Life Index (QL-Index)

QL-Index, which has 5 items, was developed by Spitzer et al. [6]. It was widely used to assess the overall QOL of cancer patients from patient's activities, daily life, health, support, and overall situation. Each item uses a 2-point scale (0–2), and the total scale is ranging from 0 to 10. A high score means a healthy or high QOL [6, 7].

#### 2.3.2. European Organization for Research and Treatment Quality of Life Version 3.0 Questionnaire (EORTC QLQ-C30)

EORTC QLQ-C30 is a multidimensional questionnaire designed for measuring the QOL of patients who have cancer. The questionnaire is made up of 30 items and includes 5 functional scales (physical, cognitive, emotional, role, and social), 3 symptom scales (pain, fatigue, and nausea and vomiting), a number of single items (dyspnea, insomnia, appetite loss, diarrhea, and constipation), financial impact, and global QOL. Most items use a 4-point scale (from 1 (not at all) to 4 (very much)). Raw scores are transformed to a 0–100 scale, and a high score means a healthy or high global health status [8–10].

In the follow-up period, patients were asked to fulfill the above two questionnaires six months after the operation.

### 2.4. Statistical Analysis

Statistical analysis was performed using the SPSS Version 19.0 (IBM, USA). Student's *t*-test, Fischer's exact probability test, and Pearson's *χ*^2^ were used to examine differences between the elderly and nonelderly groups. Continuous variables were presented as means ± SD. A *P* value <0.05 was considered statistically significant.

## 3. Results

The baseline and clinicopathological characteristics of the elderly and nonelderly patients are summarized in Table 1. The median age in the elderly group was 72.6 ± 3.6 years; in the nonelderly group, the number was 65.8 ± 9.2. The gender ratio, body mass index (BMI), location endoscopic classification, and pathological pattern were not significantly different between the two groups. The size of the lesion was measured in the longest axis. No difference was found in the size of the lesions between the two groups (29.7 ± 7.6 mm versus 28.7 ± 6.8 mm, *P* = 0.643). Similarly, there were no significant differences in mean procedure time, hospitalization days, complete resection, and en bloc resection.

As shown in Table 2, there were no significant differences between the two groups in the complications observed during ESD procedure. Significant bleeding during ESD occurred in only 1 case in the elderly group and 2 cases in the nonelderly group, which successfully disposed with endoscopic hemostasis. One patient (0.9%) in the elderly group and 4 nonelderly patients encountered the perforation, the episodes were all solved very well with clipping, and no cases required surgical intervention. When the pulse rate was under 40 beats per minute with vasovagal reflex reaction, we use deaeration with endoscopy. If the pulse did not improve, we administered atropine sulfate to deal with the bradycardia. Oxygen administration was performed if the decreasing in oxygen concentration happened.

Complications observed after ESD procedure are shown in Table 3. Of the 109 elderly patients, 3 patients (2.8%) underwent the postoperative bleeding. In the nonelderly group, the occurrence rate was 2.6% (7/270), no difference was found between the two groups (*P* = 0.596). All patients with postoperative bleeding required a repeat colonoscopy, and they were all treated by using endoscopic approach to stop bleeding. Only 1 patient in the elderly group needed a blood transfusion. The occurrence rate of postoperative perforation was 0.9% in the elderly group and 0.4% in the nonelderly, respectively (*P* = 0.265), and all of them were well treated. With respect to the pyrexia (defined as temperature above 38.0°C regardless of its duration), no difference was found between the two groups (*P* = 0.902). Two patients in the elderly group and 2 patients in the nonelderly group were diagnosed with pneumonia, and no statistical difference was found (0.9% versus 1.1%, *P* = 0.546).

QL-Index and EORTC QLQ-C30 questionnaires were performed at sixth month post-ESD. All patients completed the questionnaires.  Table 4 illustrates that the scores of the total score of QL-Index and the other five categories showed no statistical difference between the elderly group and nonelderly group. For EORTC QLQ-C30, the global QOL score in the elderly group was 72.1 ± 21.4; for the nonelderly group, it was 74.9 ± 19.3 and no statistical difference was found (Table 5). Similarly, there were no significant differences in functional scale and symptom scale.

## 4. Discussion

Elderly patients with systemic and chronic diseases, together with poor gastrointestinal function, might not be able to endure severe surgical trauma. Several studies showed that ESD procedure did not change the normal anatomic structure and physiological function of gastrointestinal tract, and it displayed lower complication and mortality rates than surgery [11–14]. In recent years, ESD was regarded as the first-line treatment for early-stage gastrointestinal tumors [12]. To our knowledge, studies reporting the treatment of ECC by ESD in elderly patients are lacking in China. In this study, the findings indicate that ESD was relatively safe and effective for elderly patients with ECC, and the postoperative overall quality of life in elderly patients was equal to the nonelderly patients.

In our study, we demonstrated that there was no significant difference in the mean procedure time between elderly and nonelderly patients, which is in line with a study from Japan [3]. Similarly, there were no significant differences in hospitalization days, complete resection, and en bloc resection between the two groups. In our research, because the selection bias may be involved, we did not compare the medical costs between elderly and nonelderly patients. Higher complete resection or en bloc resection means lower tumor recurrence [15]. Researches indicated that the complete resection rate and en bloc resection rate were above 90% in the treatment of early stage gastrointestinal tumors [16, 17]. Our result demonstrated that the complete resection rate and en bloc resection rate were 93.0% and 94.7% in the elderly patients. Predictably, the 5-year survival rate of these elderly patients will be high.

ESD is often associated with high frequencies of intraoperative and postoperative complications [18]. In this study, there were no significant differences between the elderly and nonelderly patients in the complications observed during and after ESD procedure. Only 1 case in the elderly group occurred significant bleeding during ESD, and 3 elderly patients encountered it after ESD procedure. We successfully disposed this complication with endoscopic methods. Continuous use of anticoagulant drugs, histology, invasion depth of tumor, and larger lesions was considered as possible reasons for the significant bleeding during ESD [19–21]. Lesions located in the cecum, significant bleeding during the ESD procedure, and without good at disposing the exposed blood vessels may increase the incidence of postoperative bleeding [4, 18, 21]. Second-look endoscopy was considered as an effective method for preventing postoperative bleeding; however, it is still controversial [22].

Perforation is the most serious complication in colorectal ESD [4]. It occurred in 1 elderly patient during ESD and 1 elderly patient after ESD procedure. The rate of this complication varies between 1% and 10% in experienced centers in Japan [4, 23]. In most previous cases, perforation was treated conservatively [3]; similarly, in this study, the episode was solved very well with clipping, and no case required surgical intervention. In most previous studies, larger lesion (>20 mm), hypertension, massive submucosal invasion, tumor location, and lack of experience in ESD were deemed to be the risk factors for perforation during and after ESD [3, 24].

In the present study, the incidence of “bradycardia” (<40 beats per minute) and “decrease in oxygen concentration” that occurred in the elderly group was 12.8% and 34.9%, which is similar with a report from Japan [3]. To our knowledge, the two complications during ESD have not been investigated in China. In our ESD practice, the above two complications were common. Without timely and appropriate management, they will greatly affect the progress of ESD. Deaeration and administered atropine sulfate are good and effective methods to deal with the bradycardia. Oxygen administration was performed in our ESD procedure if the decrease in oxygen concentration happened.

As far as we know, no previous report has described the pyrexia (defined as a body temperature above 38.0°C, regardless of its duration) after colorectal ESD procedure. Several previous researches aiming at gastric ESD suggested that pyrexia may relate to many other complications, especially pneumonia, so we should pay more attention to this complication [25, 26]. Because there were differences between participants, treatment methods, and devices, the results of the previous studies focusing on the incidence of the pyrexia which happened after gastric ESD vary from 2% to 24.8% [25–27]. Our study showed that the incidence of the pyrexia was relatively low in the elderly group (3.7%, 4/109). One patient evolved into pneumonia, and 3 patients rehabilitated with nondrug ways. The exact risk factors for pyrexia are largely unknown; longer procedure time, larger resection size, and proficiency of the operator may be involved. Watari et al. said that 66.7% of the patients with CT-founded pneumonia after ESD had no abnormal findings on plain chest radiography [28]. In this study, we diagnosed pneumonia by chest CT, and the pneumonia occurred in 1 elderly patient (0.9% (1/109)). We treated the pneumonia successfully by using antibiotics. For elderly patient, it is necessary to conduct researches to determine if pyrexia is a risk factor for pneumonia. At the same time, the standard ESD procedure does not include antibiotic prophylaxis. Whether the antibiotic prophylaxis is a necessity for preventing pyrexia deteriorated into pneumonia after ESD is worth researching.

Assessment of QOL of the elderly patients with ECC who underwent the ESD procedure to care and monitor for both psychological and medical consequences of their ESD treatment and rehabilitation is imperative. Based on our findings, there were no statistically significant differences in QL-Index and EORTC QLQ-C30 scores between elderly and nonelderly patients performed at sixth month post-ESD. Our survey suggests that the global QOL of the elderly patients was comparability.

Some limitations should be considered. One limitation is that we did not perform a randomized trial comparing the elderly and nonelderly patients. It was theoretically feasible, but this would not conform to the principles of medical ethics. Second, it was a retrospective study from a single center; however, the enough sample size may ensure the credibility of our results. Third, our follow-up time was not long enough; the global QOL of the elderly patients with long-term assessment after ESD procedure should be evaluated. Further study should pay more attention to multicentric and prospective research with a larger number of subjects and longer follow-up time.

## 5. Conclusions

These findings indicate that compared with nonelderly patients, ESD was relatively safe and effective for elderly patients. At the same time, the postoperative overall quality of life in elderly patients was equal to the nonelderly patients. ESD may be a recommended first-line treatment for ECC in elderly patients.

## Figures and Tables

**Table 1:** Patients' baseline and clinicopathological characteristics in the elderly and nonelderly groups.

	Elderly group (*n* = 109)	Nonelderly group (*n* = 270)	*P* value
Gender ratio (M : F)	60/49	158/112	0.309
Age (year)	72.6 ± 3.6	65.8 ± 9.2	
BMI	22.4 ± 3.2	22.6 ± 3.7	0.873
Location (*n*)			0.241
Rectum	39	94	
Sigmoid colon	26	73	
Descending colon	24	68	
Transverse colon	18	38	
Ascending colon	6	15	
Lesions (*n*)	113	288	
pT stage			0.282
Tis	76	202	
T1a	33	86	
Endoscopic classification			0.307
I	45	104	
II	60	146	
LST	8	38	
Pathological pattern (*n*)			0.276
Papillary adenocarcinoma	52	124	
Tubular adenocarcinoma	45	99	
Mucous adenocarcinoma	13	49	
Signet ring cell carcinoma	1	4	
Others	2	12	
Lesion size (mm)	29.7 ± 7.6	28.7 ± 6.8	0.643
Mean procedure time (min)	56.1 ± 12.9	55.9 ± 14.0	0.672
Hospitalization days	5.9 ± 2.4	5.4 ± 2.6	0.658
Complete resection	105/113 (93.0%)	273/288 (94.8%)	0.347
En bloc resection	107/113 (94.7%)	278/288 (96.5%)	0.312

BMI: body mass index; LST: laterally spreading tumor: “Others” means undifferentiated carcinoma, squamous carcinoma, adenosquamous carcinoma, clear cell carcinoma, neuroendocrine carcinoma, and so forth.

**Table 2:** The complications observed during ESD procedure.

	Elderly group (*n* = 109)	Nonelderly group (*n* = 270)	*P* value
Significant bleeding during ESD	1 (0.9%)	2 (0.7%)	0.629
Perforation	1 (0.9%)	4 (1.5%)	0.147
Bradycardia (<40 beats per minute)	14 (12.8%)	30 (11.1%)	0.168
Decrease in oxygen concentration (Sat O_2_ < 90%)	38 (34.9%)	86 (31.9%)	0.152

**Table 3:** The complications observed after ESD procedure.

	Elderly group (*n* = 109)	Nonelderly group (*n* = 270)	*P* value
Postoperative bleeding	3 (2.8%)	7 (2.6%)	0.596
Postoperative perforation	1 (0.9%)	1 (0.4%)	0.265
Pyrexia	4 (3.7%)	10 (3.7%)	0.902
Pneumonia	1 (0.9%)	3 (1.1%)	0.546

**Table 4:** The QL-Index scores between elderly and nonelderly groups.

	Number (*n*)	Activities	Daily life	Health	Support	Overall situation	Total score
Elderly group	109	1.52 ± 0.45	1.56 ± 0.52	1.42 ± 0.47	1.37 ± 0.28	1.52 ± 0.39	7.44 ± 1.95
Nonelderly group	270	1.70 ± 0.46	1.65 ± 0.25	1.46 ± 0.28	1.45 ± 0.33	1.58 ± 0.37	7.80 ± 1.83
*P* value		0.416	0.502	0.663	0.627	0.764	0.562

**Table 5:** The EORTC QLQ-C30 scores between elderly and nonelderly groups.

	Elderly group (*n* = 109)	Nonelderly group (*n* = 270)	*P* value
Functional scale			
Physical function	77.1 ± 22.4	79.5 ± 18.7	0.618
Performing roles	92.7 ± 23.1	92.1 ± 19.3	0.806
Emotional function	83.1 ± 27.9	87.0 ± 25.7	0.589
Cognitive function	70.3 ± 25.9	71.4 ± 27.0	0.738
Social function	65.1 ± 20.4	64.2 ± 25.6	0.720
Symptoms scale			
Pain	20.7 ± 5.5	21.0 ± 4.9	0.671
Fatigue	25.5 ± 4.3	25.8 ± 6.6	0.847
Nausea and vomiting	20.3 ± 6.6	21.4 ± 5.5	0.583
Insomnia	39.7 ± 10.4	38.1 ± 6.7	0.548
Dyspnea	16.0 ± 5.0	16.5 ± 6.2	0.728
Constipation	41.1 ± 11.4	38.0 ± 9.7	0.305
Diarrhea	24.6 ± 6.4	25.3 ± 8.6	0.752
Appetite loss	29.0 ± 9.4	29.8 ± 10.6	0.677
Financial impact	35.2 ± 12.1	36.2 ± 11.2	0.563
Global QOL	72.1 ± 21.4	74.9 ± 19.3	0.593
